# Lactose and benign ovarian tumours in a case–control study

**DOI:** 10.1054/bjoc.2000.1476

**Published:** 2000-11-22

**Authors:** J A Britton, C Westhoff, G R Howe, M D Gammon

**Affiliations:** Division of Environmental Health Science, Mt. Sinai School of Medicine, 1 Gustave L. Levy Place, Box 1057, New York, NY, 10029; Department of Obstetrics & Gynecology, College of Physicians and Surgeons, Columbia University, New York, NY, 10032; Division of Epidemiology, Joseph L. Mailman School of Public Health, Columbia University, New York, NY, 10032; Department of Epidemiology, School of Public Health, University of North Carolina, Chapel Hill, NC, 27599

**Keywords:** case–control studies, diet, lactose, nutrition, ovarian neoplasms, risk factors

## Abstract

We investigated the relation between benign ovarian tumours and lactose among 746 case women identified at seven New York metropolitan hospitals and 404 community controls, age and hospital frequency matched to the expected case distribution. No increase in risk was found for lactose (highest quartile versus lowest: adjusted odds ratio = 0.82 (95% CI 0.57–1.20) or for any other lactose foods. © 2000 Cancer Research Campaign http://www.bjcancer.com


British Journal of Cancer (2000) 83(11), 1552–1555
© 2000 Cancer Research Campaign
doi: 10.1054/ bjoc.2000.1476, available online at http://www.idealibrary.com on IDEAL®
http://www.bjcancer.com

Short Communication

### Lactose and benign ovarian tumours in a case–control study

**JA Britton[1], C Westhoff[2,3], GR Howe[3] and MD Gammon[4]**

[1]Division of Environmental Health Science, Mt. Sinai School of Medicine, 1 Gustave L. Levy Place, Box 1057, New York, NY 10029;
[2]Department of Obstetrics & Gynecology, College of Physicians and Surgeons, Columbia University, New York, NY 10032; [3]Division of Epidemiology, Joseph L. Mailman School of Public Health, Columbia University, New York, NY 10032; [4]Department of Epidemiology, School of Public Health, University of North Carolina, Chapel Hill, NC 27599

Summary We investigated the relation between benign ovarian tumours and lactose among 746 case women identified at seven New York metropolitan hospitals and 404 community controls, age and hospital frequency matched to the expected case distribution. No increase in risk was found for lactose (highest quartile versus lowest: adjusted odds ratio = 0.82 (95% CI 0.57–1.20) or for any other lactose foods. © 2000 Cancer Research Campaign http://www.bjcancer.com

**Keywords:** case–control studies; diet; lactose; nutrition; ovarian neoplasms; risk factors

Galactose levels are determined by dietary sources (primarily lactose) and metabolism-related factors. The theory linking galactose to ovarian cancer aetiology originates from galactosaemia, which is characterized by the absence of transferase. Some galactosaemics experience ovarian failure or have elevated gonadotropins levels (Kaufman et al, 1981), which may increase ovarian cancer risk (Gardner, 1961; Cramer and Welch, 1983). An increased ovarian cancer risk has been reported with higher lactose intakes and with a higher lactose to transferase activity ratio (Cramer et al, 1989).

The surgical diagnosis of benign epithelial tumours declines at ages when epithelial ovarian cancer incidence increases, suggesting that a small proportion of BOTs may progress to their invasive malignant counterparts (Bennington et al, 1968). This is consistent with observations of benign neoplasia located adjacent to or within ovarian cancers (McKay, 1962; Puls et al, 1992), and of benign to malignant epithelium histologic transition in one-quarter of a sample of ovarian cancers (Puls et al, 1992). Thus, benign and malignant ovarian tumours may share a common aetiology, and if so, they afford an opportunity to investigate potential risk factors closer to the time of aetiologic interest.

### MATERIALS AND METHODS

Methods of this study have been described in more detail elsewhere (Westhoff et al, 2000). In this study English-speaking women, aged 18 to 74, with a telephone, residing in the New York metropolitan area within 50 miles of a participating hospital, having an ovary and not having a malignant tumour were eligible. Institutional review boards approved the study protocols.

*Received 5 April 2000*
*Revised 2 August 2000*
*Accepted 3 August 2000*

*Correspondence to:* JA Britton

BOT cases were diagnosed in 1992 and 1993. A uniform pathology review determined eligibility and histologic classification (Russell and Bannatyne, 1989). Controls, frequency-matched to the expected case distribution by 10-year age group and hospital, were identified using Waksberg's random digit dialing (RDD) method (Waksberg, 1978; Hartge et al, 1984). Participation rates among the cases and controls were 80.7% ($n$ = 746) and 71.4% ($n$ = 404), respectively. RDD screener response rate was 84.9%.

A structured questionnaire was administered. A 127-item Willett food frequency questionnaire (FFQ) (Willett, 1990) about usual dietary intake during the 12 to 24 months before interview was self-completed by 90% ($n$ = 673) of the cases and 87% ($n$ = 352) of the controls (Britton et al, 2000). Primary lactose foods included skim or low-fat milk, whole milk, cream, sour cream, sherbet or ice milk, ice cream, yogurt, cottage or ricotta cheese, cream cheese, and other cheeses such as American or cheddar cheese.

Median lactose intakes were compared by the Wilcoxon test (Conover, 1980). Unconditional logistic regression produced adjusted odds ratios (ORs) and 95% confidence intervals (CIs) (Hosmer and Lemeshow, 1989). Controls were compared to all cases and to the more common histologic sub-types: endometriomas, serious adenomas and teratomas.

Lactose (grams per day) was considered as a continuous and categorical variable (classified into quartiles). The residual nutrient method was used for the latter (Willett et al. 1997). Foods were divided into three categories according to the control frequency distribution. In the models, categorical variables were represented as indicator variables and adjustment was made for age (<25/25–34/35–44/45–54/55–64/65+ years), hospital (seven categories), total energy (kilocalories per day), and body mass index (BMI: weight in kilograms/height in metres squared) for the year prior to interview. When dietary fat and non-dietary factors were considered as confounders, estimates were unaffected thus subsequent models omitted these factors. To assess trends, quartile levels or indicator variable scores were entered in models as ordinal variables.

**Table 1** Odds ratios and 95% confidence intervals for benign ovarian tumours, according to lactose intake as categorized by quartiles, among 1015 women in the New York Metropolitan Area, 1992–1993

| | Controls | Endometrioma[b] | | | Mucinous adenoma | | | Serous adenoma | | | Teratoma[b] | | | All cases[b] | | |
|---|---|---|---|---|---|---|---|---|---|---|---|---|---|---|---|---|
| **Exposure** | **(no.)[a]** | **(no.)** | **OR[a,c]** | **95% CI[a]** | **(no.)** | **OR[c]** | **95% CI** | **(no.)** | **OR[c]** | **95% CI** | **(no.)** | **OR[C]** | **95% CI** | **(no.)** | **OR[c]** | **95% CI** |
| Quartiles of lactose intake (grams) | | | | | | | | | | | | | | | | |
| Q1 (≤5.58) | 86 | 72 | 1.00 | | 15 | 1.00 | | 51 | 1.00 | | 38 | 1.00 | | 174 | 1.00 | |
| Q2 (5.59–10.42) | 87 | 77 | 1.11 | 0.69–1.79 | 14 | 0.97 | 0.41–2.28 | 30 | 0.61 | 0.34–1.10 | 36 | 1.01 | 0.56–1.81 | 161 | 0.93 | 0.63–1.36 |
| Q3 (10.43–17.11) | 88 | 74 | 1.06 | 0.66–1.70 | 20 | 1.37 | 0.62–3.02 | 51 | 1.08 | 0.63–1.83 | 50 | 1.37 | 0.79–2.38 | 185 | 1.04 | 0.71–1.52 |
| Q4 (>17.11) | 86 | 57 | 0.77 | 0.48–1.26 | 11 | 0.75 | 0.31–1.79 | 40 | 0.78 | 0.45–1.35 | 41 | 1.05 | 0.61–1.83 | 148 | 0.82 | 0.57–1.20 |
| *P* for trend | | | 0.31 | | | 0.77 | | | 0.82 | | | 0.60 | | | 0.46 | |

[a]no., number of subjects; OR, Odds ratio; CI, Confidence interval. [b]One woman with a teratoma and one with an endometrioma with missing information on body mass index (weight in kilograms/height in metres squared) were excluded from the logistic models. [c]Adjusted for age, hospital, total caloric intake, and body mass index for the year prior to interview.

**Table 2** Odds ratios and 95% confidence intervals for benign ovarian tumours in relation to lactose-food items, among 1015 women in the New York Metropolitan Area, 1992–1993

| | Controls | Endometrioma[b] | | | Serous adenoma | | | Teratoma[b] | | | All cases[b] | | |
|---|---|---|---|---|---|---|---|---|---|---|---|---|---|
| **Exposure** | **(no.)[a]** | **(no.)** | **OR[a,c]** | **95% CI[a]** | **(no.)** | **OR[c]** | **95% CI** | **(no.)** | **OR[c]** | **95% CI** | **(no.)** | **OR[c]** | **95% CI** |
| Whole milk (8 oz or 236.8 ml)[a] | | | | | | | | | | | | | |
| Never or <1/month | 191 | 168 | 1.00 | | 115 | 1.00 | | 112 | 1.00 | | 429 | 1.00 | |
| 1/month–≤1/week | 75 | 58 | 0.81 | 0.54–1.24 | 20 | 0.51 | 0.29–0.91 | 23 | 0.48 | 0.28–0.83 | 112 | 0.68 | 0.48–0.95 |
| 2+/week | 68 | 47 | 0.82 | 0.52–1.28 | 24 | 0.69 | 0.39–1.20 | 26 | 0.59 | 0.34–1.01 | 102 | 0.69 | 0.48–0.99 |
| Not ascertained | 13 | 7 | | | 13 | | | 4 | | | 25 | | |
| *P* for trend | | | 0.29 | | | 0.07 | | | 0.01 | | | 0.02 | |
| Skim/low-fat milk (8 oz or 236.8 ml) | | | | | | | | | | | | | |
| Never or <1/month | 132 | 90 | 1.00 | | 61 | 1.00 | | 50 | 1.00 | | 224 | 1.00 | |
| 1/month–≤1/week | 57 | 48 | 1.26 | 0.77–2.06 | 26 | 0.99 | 0.55–1.78 | 28 | 1.28 | 0.72–2.28 | 102 | 1.03 | 0.69–1.53 |
| 2+/week | 152 | 137 | 1.25 | 0.86–1.81 | 79 | 1.13 | 0.74–1.75 | 84 | 1.43 | 0.92–2.22 | 328 | 1.21 | 0.90–1.63 |
| Not ascertained | 6 | 5 | | | 6 | | | 3 | | | 14 | | |
| *P* for trend | | | 0.26 | | | 0.56 | | | 0.11 | | | 0.20 | |
| Yogurt (1 c or 226.8 g)[a] | | | | | | | | | | | | | |
| Never or <1/month | 116 | 94 | 1.00 | | 51 | 1.00 | | 60 | 1.00 | | 222 | 1.00 | |
| 1/month–≤ 1/week | 126 | 120 | 1.15 | 0.79–1.70 | 64 | 1.25 | 0.77–2.01 | 60 | 0.89 | 0.56–1.39 | 262 | 1.08 | 0.79–1.48 |
| 2+/week | 101 | 65 | 0.84 | 0.54–1.30 | 52 | 1.20 | 0.73–1.98 | 44 | 0.79 | 0.49–1.29 | 172 | 0.86 | 0.61–1.21 |
| Not ascertained | 4 | 1 | | | 5 | | | 1 | | | 12 | | |
| *P* for trend | | | 0.52 | | | 0.48 | | | 0.35 | | | 0.44 | |

[a]no., number of subjects; OR, odds ratio; CI, confidence interval; ml, millilitres; g, grams; oz, ounces; c, cups. [b]One woman with a teratoma and one with an endometrioma with missing information on body mass index (weight in kilograms/height in metres squared) were excluded from the logistic models. [c]Adjusted for age, hospital, total caloric intake, and body mass index for the year prior to interview.

### RESULTS

After exclusion of 1% of cases and controls with extreme energy intake (Howe et al, 1990; Hunter et al, 1996) 668 case women and 347 control women remained. Women could have multiple tumours of differing histology as a result the cases were diagnosed with 717 BOTs: 172 serous, 60 mucinous, 280 endometrioid, and 8 Brenner tumours, as well as 165 teratomas and 32 fibroma-thecomas. All women (Westhoff et al, 2000) and those providing dietary information (Britton et al, 2000) had similar distributions of demographic and other characteristics. In general, controls were significantly more likely than cases to be parous and to have a non-private or no health care provider, a possible indicator of less diagnosis opportunity. Cases were non-significantly more likely to be white, never OC users and have larger BMI. The mean case age of 42.2 years (standard deviation (SD) = 11.9) was slightly older than the mean control age of 41.5 years (SD = 12.5) ($P = 0.4$).

All cases combined and each histologic type, except endometriomas, had non-significantly higher median lactose intakes than controls (data not shown). There was no evidence of an association or a dose–response relation between lactose intake and BOTs or any of the histologic sub-types (Table 1). Continuous lactose measures yielded similar findings; the ORs and 95% CIs per 10 grams of lactose were 0.90 (0.76–1.06), 0.89 (0.65–1.21), 0.96 (0.80–1.16), 1.07 (0.89–1.27), and 0.97 (0.85–1.10), for endometriomas, mucinous adenomas, adenomas, teratomas, and all BOTs combined, respectively.

Only whole milk was associated with BOTs (Table 2). A significant inverse relation was observed for all BOTs combined and for teratoma tumours, while a borderline significant inverse association was noted for tumours. Though these tests are indicative of an inverse trend, the observed association for the middle category of whole milk intake was either the same as or stronger than that observed for the highest category of intake. There were no other statistically significant associations or dose–response

© 2000 Cancer Research Campaign 

relations for BOTs combined or for the individual histologic sub-types and consumption of any other lactose foods (selected items shown in Table 2).

### DISCUSSION

In this study, whole milk was the only item significantly associated with BOTs for which estimates were below the null. Adjustment for total and types of dietary fat as well as lactose did not change the association. Thus, our results do not support an increased BOT risk in relation to the lactose or dietary fat component of dairy products. This agrees with our earlier finding of no relation between BOTs and saturated fat (Britton et al, 2000).

If BOTs share a common aetiology with, or are precursors of malignant tumours, then the suggestion that either lactose or high-fat dairy products (Mettlin and Piver, 1990) increase ovarian cancer risk is not supported by this study. Our null lactose findings are consistent with studies examining borderline (Risch et al, 1996) or malignant ovarian (Engle et al, 1991; Risch et al, 1994a; Herrinton et al, 1995; Mink et al, 1996; Webb et al, 1998) tumours, but contrast the findings of an elevated ovarian cancer risk in relation to lactose intake (Cramer et al, 1989) or in relation to the lactose to transferase ratio (Cramer et al, 1989). Lactose consumption relative to metabolic capability may be a more relevant measure of galactose exposure but information on transferase activity or lactose tolerance was not available. Finally, we found a reduced BOT risk associated with higher whole milk consumption. Studies of ovarian cancer risk and either whole milk (Cramer et al, 1984, 1989; Mettlin and Piver, 1990; Ursin et al, 1990; Risch et al, 1994a; Webb et al, 1998; Kushi et al, 1999) or dietary fat (Byers et al, 1983; Shu et al, 1989; Slattery et al, 1989; Tzonou et al, 1993; Rische et al, 1994b; Mink et al, 1996; Webb et al, 1998; Kushi et al, 1999) consumption have inconsistent findings, generally reporting no association or an elevated risk.

Participants in health-related studies might be more health conscious and therefore more likely to consume or report low-fat foods. This, coupled with the lower control response rate, could result in selection bias. Or, cases may be more motivated to provide truthful responses than controls, resulting in recall bias. These biases would result in an underestimation of low-fat, but an overestimation of high-fat, food associations. In light of the null findings, it is hard to conceive that these biases are selectively affecting low-fat food associations. Lactose findings should be unaffected because the lactose and fat content of foods are independent.

We assessed commonly eaten major and minor lactose sources enabling us to rank participants' lactose exposure. The foods assessed were similar to the short list of items examined in a study reporting a high correlation (r = 0.96) between lactose estimated using 34 versus 7 lactose foods (Cooper et al, 1995). Among controls the expected ethnic/racial variation in lactose intake was observed (Scrimshaw and Murray, 1988). These findings, together with the similar mean lactose intakes for our white controls and those in another study (Cramer et al, 1989), lend credence to our lactose measure.

Overall the study's findings do not support an elevated BOT risk in relation to lactose and are consistent with the results of most of the ovarian cancer studies (Engle et al, 1991; Risch et al, 1994a, 1996; Herrinton et al, 1995; Mink et al, 1996; Webb et al, 1998). The failure to detect an association might reflect a lack of power particularly in the histologic sub-type analyses. Finally, the reduction in BOT risk for greater whole milk intake could be a chance finding given the multiple comparisons made.

### ACKNOWLEDGEMENTS

Study funding was provided by National Cancer Institute grant #CA50658. The authors would like to acknowledge the work of Dr Tom Wright, the study pathologist, who reviewed all pathology reports referencing the ovary to determine potentially eligible women. A second review of the slides was conducted to verify eligibility and to classify the tumour histology type.

### REFERENCES

Bennington JL, Ferguson BR and Haber SL (1968) Incidence and relative frequency of benign and malignant ovarian neoplasms. *Obstet Gynecol* **32**: 627–632

Britton JA, Westhoff C, Howe GR and Gammon MD (2000) Diet and benign ovarian tumors. *Cancer Causes & Control* **11**: 389–401

Byers T, Marshall J, Graham S, Mettlin C and Swanson M (1983) A case-control study of dietary and nondietary factors in ovarian cancer. *J Natl Cancer Inst* **71**: 681–686

Conover WJ (1980) *Practical Nonparametric Statistics*. John Wiley & Sons, Inc. New York

Cooper GS, Busby MG and Fairchild AP (1995) Measurement of lactose consumption reliability and comparison of two methods. *Ann Edidemiol* **5**: 473–477

Cramer DW and Welch WR (1983) Determinants of ovarian cancer risk. II. Inferences regarding pathogenesis. *J Natl Cancer Inst* **71**: 717–721

Cramer DW, Welch WR, Hutchison GB, Willett W and Scully RE (1984) Dietary animal fat in relation to ovarian cancer risk. *Obstet Gynecol* **63**: 833–838

Cramer DW, Harlow BL, Willett WC, Welch WR, Bell DA, Scully RE, Ng WG and Knapp RC (1989) Galactose consumption and metabolism in relation to the risk of ovarian cancer. *Lancet* **2**: 66–71

Engle A, Muscat JE and Harris RE (1991) Nutritional risk factors and ovarian cancer. *Nutr Cancer* **15**: 239–247

Gardner WU (1961) Tumorigenesis in transplanted irradiated and nonirradiated ovaries. *J Natl Cancer Inst* **26**: 829–853

Hartge P, Brinton LA, Rosenthal JF, Cahill JI, Hoover RN and Waksberg JS (1984) Random digit dialing in selecting a population-based control group. *Am J Epidemiol* **120**: 825–833

Herrinton LJ, Weiss NS, Beresford SA, Stanford JL, Wolfla DM, Feng Z and Scott CR (1995) Lactose and galactose intake and metabolism in relation to the risk of epithelial ovarian cancer. *Am J Epidemiol* **141**: 407–416

Hosmer DW and Lemeshow S (1989) *Applied Logistic Regression*. John Wiley & Sons, Inc.: New York

Howe GR, Hirohata T, Hislop TG, Iscovich JM, Yuan J-M, Katsouyanni K, Lubin F, Marubini E, Modan D, Rohan T, Toniolo P and Shunzhang Y (1990) Dietary factors and risk of breast cancer: combined analysis of twelve case-control studies. *J Natl Cancer Inst* **82**: 561–569

Hunter DJ, Spiegelman D, Adami H, Beeson L, van den Brandt PA, Folsom AR, Fraser GE, Goldbohm A, Graham S, Howe GR, Kushi LH, Marshall JR, McDermott A, Miller AB, Speizer FE, Wolk A, Yaun S and Willett W (1996) Cohort studies of fat intake and the risk of breast cancer – a pooled analysis. *N Engl J Med* **334**: 356–361

Kaufman FR, Kogut MD, Donnell GN, Goebelsmann U, March C and Koch R (1981) Hypergonadotropic hypogonadism in female patients with galactosemia. *N Engl J Med* **304**: 994–998

Kushi LH, Mink PJ, Folsom AR, Anderson KE, Zheng W, Lazovich D and Sellers TA (1999) Prospective study of diet and ovarian cancer. *Am J Epidemiol* **149**: 21–31

McKay DG (1962) The origins of ovarian tumors. *Clin Obstet Gynecol* **5**: 1181–1197

Mettlin CJ and Piver MS (1990) A case-control study of milk-drinking and ovarian cancer risk. *Am J Epidemiol* **132**: 871–876

Mink P, Kushi LH, Sellers T, Sheng W and Folsom A (1996) Dietary risk factors for ovarian cancer: A prospective study of older women (Abstract). *Am J Epidemiol* **143**: S59

Puls LE, Powell DE, DePriest PD, Gallion HH, Hunter JE, Kryscio RJ and van Nagell JR (1992) Transition from benign to malignant epithelium in mucinous and serous ovarian cystadenocarcinoma. *Gynecol Oncol* **47**: 53–57

*© 2000 Cancer Research Campaign*

Risch HA, Jain M, Marrett LD and Howe GR (1994a) Dietary lactose intake, lactose intolerance, and the risk of epithelial ovarian cancer in southern Ontario (Canada). *Cancer Causes & Control* **5**: 540–548

Risch HA, Jain M, Marrett LD and Howe GR (1994b) Dietary fat intake and risk of epithelial ovarian cancer. *J Natl Cancer Inst* **86**: 1409–1415

Risch HA, Marrett LD, Jain M and Howe GR (1996) Differences in risk factors for epithelial ovarian cancer by histologic type results of a case-control study. *Am J Epidemiol* **144**: 363–372

Russell P and Bannatyne P (1989) *Surgical Pathology of the Ovaries*. Churchill Livingstone: Edinburgh

Scrimshaw NS and Murray EB (1988) The acceptability of milk and milk products in populations with a high prevalence of lactose intolerance. *Am J Clin Nutr* **48**: 1079–1159

Shu XO, Gao YT, Yuan JM, Ziegler RG and Brinton LA (1989) Dietary factors and epithelial ovarian cancer. *Br J Cancer* **59**: 92–96

Slattery ML, Schuman KL, West DW, French TK and Robison LM (1989) Nutrient intake and ovarian cancer. *Am J Epidemiol* **130**: 497–502

Tzonou A, Hsieh CC, Polychronopoulou A, Kaprinis G, Toupadaki N, Trichopoulou A, Karakatsani A and Trichopoulos D (1993) Diet and ovarian cancer: a case-control study in Greece. *Int J Cancer* **55**: 411–414

Ursin G, Bjelke E, Heuch I and Vollset SE (1990) Milk consumption and cancer incidence: a Norwegian prospective study. *Br J Cancer* **61**: 454–459

Waksberg JS (1978) Sampling methods for random digit dialing. *Journal of the American Statistical Association* **73**: 40–46

Webb PM, Bain CJ, Purdie DM, Harvey PW and Green A (1998) Milk consumption, galactose metabolism and ovarian cancer (Australia). *Cancer Causes Control* **9**: 637–644

Westhoff C, Britton JA, Gammon MD, Wright T and Kelsey JL (2000) Oral contraceptives and benign ovarian tumors. *Am J Epidemiol* **152**: 242–246

Willett W (1990) *Nutritional Epidemiology*. Oxford University Press, Inc.: New York

Willett WC, Howe GR and Kushi LH (1997) Adjustment for total energy intake in epidemiologic studies. *Am J Clin Nutr* **65**: 1220S–1228S

*© 2000 Cancer Research Campaign*

